# Exploring the role of non-coding RNAs in atrial septal defect pathogenesis: A systematic review

**DOI:** 10.1371/journal.pone.0306576

**Published:** 2024-08-22

**Authors:** Zahra AmiRsardari, Akram Gholipour, Zahra Khajali, Majid Maleki, Mahshid Malakootian

**Affiliations:** 1 Cardiogenetic Research Center, Rajaie Cardiovascular Medical and Research Center, Iran University of Medical Sciences, Tehran, Iran; 2 School of Medicine, Tehran University of Medical Sciences, Tehran, Iran; 3 Congenital Heart Disease Research Center, Rajaie Cardiovascular Medical and Research Center, Iran University of Medical Sciences, Tehran, Iran; Noakhali Science and Technology University Faculty of Pharmacy, BANGLADESH

## Abstract

**Background:**

Extensive research has recognized the significant roles of non-coding RNAs (ncRNAs) in various cellular pathophysiological processes and their association with diverse diseases, including atrial septal defect (ASD), one of the most prevalent congenital heart diseases. This systematic review aims to explore the intricate involvement and significance of ncRNAs in the pathogenesis and progression of ASD.

**Methods:**

Four databases (PubMed, Embase, Scopus, and the Web of Science) were searched systematically up to June 19, 2023, with no year restriction. The risk of bias assessment was evaluated using the Newcastle-Ottawa scale.

**Results:**

The present systematic review included thirteen studies with a collective study population of 874 individuals diagnosed with ASD, 21 parents of ASD patients, and 22 pregnant women carrying ASD fetuses. Our analysis revealed evidence linking five long ncRNAs (*STX18*-*AS1*, *HOTAIR*, *AA709223*, *BX478947*, and *Moshe*) and several microRNAs (hsa-miR-19a, hsa-miR-19b, hsa-miR-375, hsa-miR-29c, miR-29, miR-143/145, miR-17-92, miR-106b-25, and miR-503/424, miR-9, miR-30a, miR-196a2, miR-139-5p, hsa-let-7a, hsa-let-7b, and hsa-miR-486) to ASD progression, corresponding to previous studies.

**Conclusions:**

NcRNAs play a crucial role in unraveling the underlying mechanisms of ASD, contributing to both biomarker discovery and therapeutic advancements. This systematic review sheds light on the mechanisms of action of key ncRNAs involved in ASD progression, providing valuable insights for future research in this field.

## 1. Introduction

Atrial septal defect (ASD) is one of the most common congenital heart diseases (CHDs), accounting for 10%–15% of CHDs, with an estimated occurrence of 25 cases per 10,000 live births [1, 2]. This defect enables communication between the left and right atria, causing a left-to-right shunt in most cases, classifying it as an acyanotic CHD. Although most ASD cases occur sporadically, some instances exhibit familial patterns, predominantly through autosomal dominant inheritance [1]. ASD can be classified into four main types, with ostium secundum as the most prevalent (70%–80% of cases), usually occurring in isolated form. The second most frequent type is ostium primum ASD (15%–20% of cases), often associated with other cardiac anomalies like ventricular septal defect and atrioventricular anomalies. The remaining types are sinus venosus and coronary sinus defects, which are less prevalent [3]. In isolated ASD, symptoms are typically minimal until the second to fourth decades of life, and diagnosis often occurs incidentally during medical tests for unrelated issues. Nonetheless, moderate to large ASD can lead to volume overload, heart failure, and pulmonary arterial hypertension over time due to left-to-right shunting, probably resulting in symptoms like exercise intolerance, syncope, and fatigue [1].

Genetic implications in syndromic and non-syndromic ASD are unequivocal. The first mutation involved in non-syndromic ASD was reported in the homeodomain of *NKX2–5* [4]. Mutations in the *GATA* family have also been associated with ASD [5]. Additionally, mutations in α-myosin heavy chain 6 (*MYH6*) and Cardiac α-actin 1 (*ACTC1*) have been observed in familial ASD [6, 7].

In recent decades, studies have indicated that, rather than gene mutations, alterations in gene expression at the transcriptome level may play a crucial role in the development of ASD, contributing significantly to the pathogenesis of the disease. Transcriptomic studies have revealed the involvement of thousands of non-coding RNAs (ncRNAs) in modulating gene expression. NcRNAs are functional RNA molecules that play essential biological roles without encoding proteins [8]. They exert influence through diverse mechanisms, including transcriptional and post-transcriptional regulation and epigenetic modifications. These transcripts come in various forms, such as long non-coding RNAs (lncRNAs) and short ncRNAs, including microRNAs (miRNAs), PIWI-interacting RNA, and small interfering RNAs [9–11]. LncRNAs, exceeding 200 nucleotides in length, generally regulate transcriptional and epigenetic processes [12]. On the other hand, miRNAs are small, single-stranded ncRNAs consisting of between 21 and 23 nucleotides. They are regulatory elements for post-transcriptional gene expression by degrading mRNAs or inhibiting mRNA translation [13].

In this systematic review, our primary aim is to conduct a comprehensive review of ncRNAs associated with ASD development. We also intend to delve into their potential functions and investigate the signaling pathways implicated in ASD progression. By doing so, we aspire to enhance the comprehension of the underlying pathogenesis of ASD and pave the way for future research in this field.

## 2. Methods

### 2.1. Search strategy and study selection

Our systematic review was conducted in accordance with the guidelines provided by the Preferred Reporting Items for Systematic Reviews and Meta-analysis (PRISMA) 2020 statement [14]. Four databases (PubMed, Embase, Web of Science, and Scopus) were searched systematically up to June 19, 2023, without any time restriction. All relevant articles up to that date were considered for potential inclusion in this study. The main search terms utilized were "non-coding RNA*", "non-protein coding RNA*", "untranslated RNA*", "congenital heart defect*", "atrial septal defect*", and "heart septal defect*". Detailed search queries can be found in S1 Table. The protocol for this systematic review was registered in PROSPERO in August 2023 (registration code: CRD42023439213).

Exploration of the expression or role of ncRNAs in ASD was the inclusion criterion in this review. Conference abstracts, protocols, and review articles were not considered eligible studies for inclusion. Moreover, the lists of references from eligible studies and relevant review articles were checked carefully to find additional relevant studies that might not have appeared in our initial database searches. All records identified in the search were imported into an EndNote library, and duplicates were removed subsequently. Two independent reviewers (ZA and AGH) initially screened all the studies based on titles and abstracts and performed a comprehensive review of the full texts.

**Fig 1:**
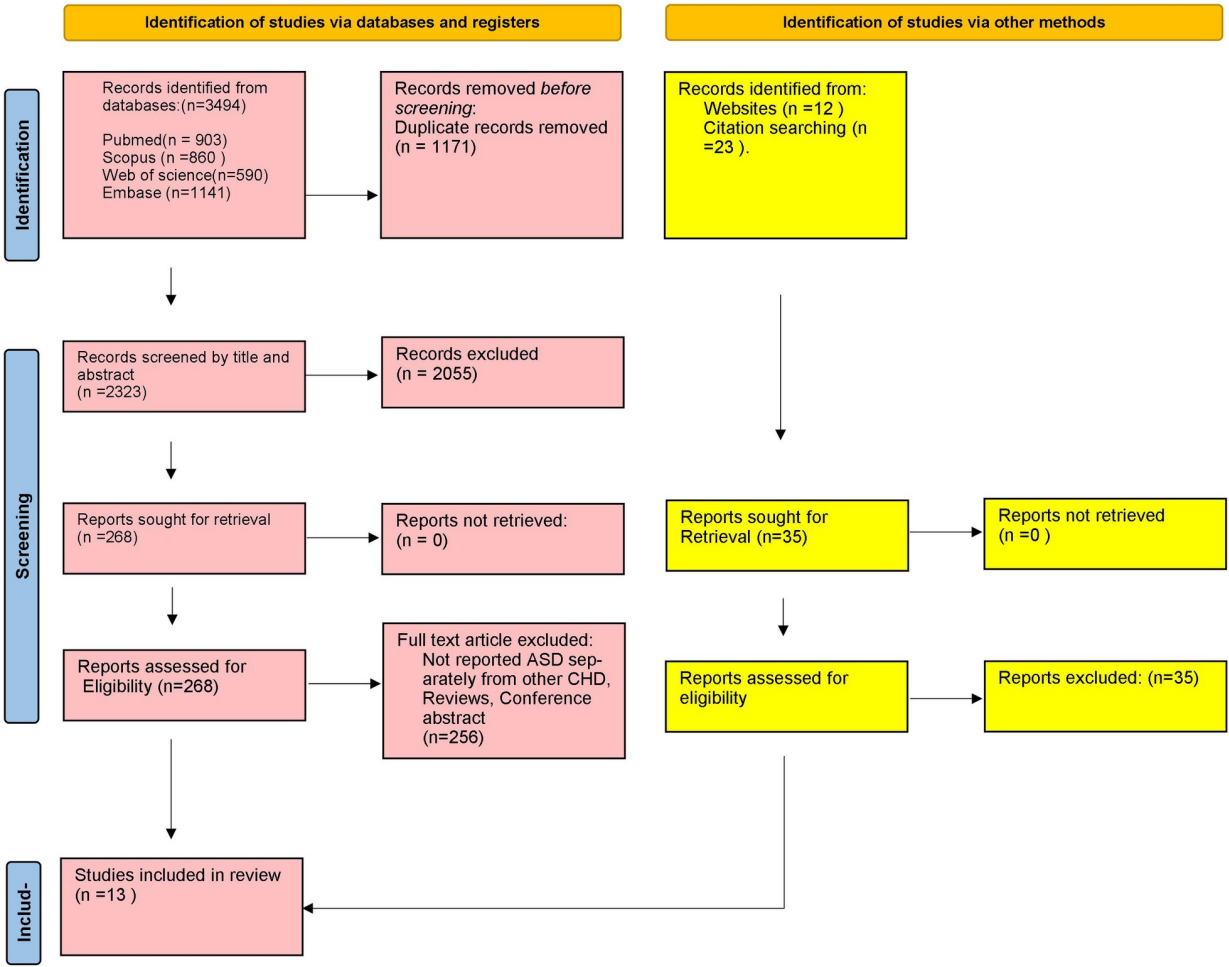
PRISMA flowchart of study selection and screening.

Whenever disagreements arose, a third reviewer (MM) expertly intervened to resolve discrepancies and finalize the selection of the included studies. Fig 1 presents a concise overview of the process used to identify and select the studies.

### 2.2. Quality assessment and data extraction

Data extraction from the included studies was executed using the Microsoft Excel Spreadsheet software. In this two-step approach, one reviewer (ZA) undertook the initial extraction of pre-specified data, while a second reviewer (AGH) performed a thorough cross-verification of the extracted data. Discrepancies were resolved through discussion in pairs (ZA and AGH) and with a third author (MM) when necessary. The extracted data encompassed various vital aspects, including 1) article title, first author’s name, publication year, country of publication, and study design; 2) study population, baseline demographic characteristics, and type of ASD; 3) name and type of discovered ncRNAs; 4) level of in vivo/in vitro expression of ncRNAs; 5) target genes and effects of ncRNAs on their target genes; and 6) other principal findings.

Two independent reviewers (ZA and AGH) conducted a thorough evaluation of the quality of each included study. This evaluation was based on the Newcastle-Ottawa scale (NOS), a well-established method for assessing the quality of observational studies [15]. Disagreements were addressed by a third reviewer (MM) to ensure consensus. The NOS employs a scoring system that spans from 0 to 9, with studies receiving a score of 6 or higher classified as having a high level of quality.

## 3. Results

### 3.1. Characteristics of the included studies

The initial search yielded 3494 results, with contributions from four databases: 903 from PubMed, 1141 from Embase, 590 from the Web of Science, and 860 from Scopus. Subsequently, duplicate entries were removed meticulously (n = 1171), leaving 2323 unique studies to be screened further based on their titles and abstracts. Following this screening stage, 268 studies were subjected to a comprehensive evaluation through full-text screening. Subsequently, thirteen studies were carefully selected for this review from database searching. An extensive website and citation search was conducted, and 35 additional studies were evaluated. Nevertheless, none met the eligibility criteria for inclusion. Finally, thirteen studies were included in this review. Fig 1 provides a detailed depiction of the selection process and reasons for exclusion. Table 1 presents a comprehensive overview of the included studies published from 2013 through 2022. Our study distribution evaluation revealed that China had the most representation, with nine studies, followed by the United Kingdom, with two. Additionally, Australia and Korea each contributed one study. The collective study population consisted of 874 individuals diagnosed with ASD, 21 parents of ASD patients, 22 pregnant women carrying ASD fetuses, and 4674 healthy controls. Out of the thirteen studies examined, six focused on the expression of lncRNAs, while the remaining seven delved into the expression of miRNAs. No study investigated other types of ncRNAs. Furthermore, among those studies, eleven were case-control investigations. The case groups encompassed ASD patients (7 studies), ASD patients along with their parents (1 study), pregnant women carrying ASD fetuses (2 studies), and ASD fetuses (1 study). Two other studies were conducted in vitro, involving work on cell lines. Out of the studies included, ten were considered to possess high quality (NOS >6), as determined by the NOS quality assessment method. The NOS criteria did not apply to two studies since they were conducted in vitro using cell lines and did not involve case and control groups. Detailed NOS scoring can be found in S2 Table.

**Table 1:** Characteristics of studies evaluating non-coding RNAs(LncRNAs / miRNAs) in ASD. (NOS: Newcastle-Ottawa scale. N/A: Not applicable).

Type of non-coding RNA	Study	year	Country	Samples	Patients(n)	Controls (n)	Non-coding RNAs	Level of expression	Target genes	Other finding	NOS
	Cordell	2013	UK	ASD patients	417	2520	STX18-AS1	Downregulated (in vitro)	-	Three ASD-associated SNPs were identified: rs6824295 (T>C), rs16835979 (A>C), and rs870142 (T>C). These three SNPs are linked to reduced expression of STX18-AS1.	6
**Long non-coding RNAs**	Gu	2016	China	Plasma samples of pregnant women with ASD fetus	18	62	AA709223, BX478947	Both downregulated	-	-	7
	Jiang	2018	China	Plasma samples of ASD patients	36	36	HOTAIR	Upregulated	-	HOTAIR has interactions with proteins including EZH1, EZH2, SUZ12, REST, KDM1A, RCOR1	7
	Li	2018	China	Plasma samples of ASD patients	103	730	MALTA1	-		MALAT1 rs619586 polymorphism minimally (not significant) increases the risk of ASD (OR = 0.83, p-value = 0.31)	7
	Kim	2021	Korea	-	-	-	Moshe	-	Moshe increases expression of NKX2-5 Silencing Moshe increased expression of Isl-1, Hand2, and Tbx2 genes	-	N/A
	Liu	2022	UK	-	-	-	STX18-AS1	-	STX18-AS1 has a trans-acting inhibitory effect on NKX2-5	-	N/A
**MicroRNAs**	Zhu	2013	China	Serum samples of pregnant women with ASD fetus	4	27	hsa-mir-19b, hsa-mir-375 and hsa-mir-29c	Upregulated	-	-	7
	Wang	2016	China	Serum samples of familal isolated ASD II patients	4	1	miR-139-5p	-	3′UTR of ACTC1	c.*1784T>C mutation in the 3′UTR of ACTC1 in familial ASD patients which results in new miR-139-5p target site, miR-139-5p binding to this target site decreases ACTC1 expression	4
	Yu	2016	China	Plasma samples of ASD patients	174	276	miR-196a2	-	-	rs11614913 (T>C) SNP of miR-196a2 is associated with ASD occurrence, and this polymorphism can increase the expression of miR-196a2 .	7
	Wang	2017	China	Blood samples of ASD patients	120	990	miR-9 and miR-30a	-	Higher Binding affinity of miR-9 and miR-30a to TBX5 3′UTR reduces TBX5 expression.	rs6489956 C>T variation of TBX5 3′UTR is associated with ASD because T allele has a higher affinity for binding to miR-9 and miR-30a compared to C allele and this increase in binding leads to a reduction in TBX5 expression	7
	Song	2018	Australia	Plasma samples of ASD patients and their parents	12 (ASD patients),21 (parents)	30	hsa-let-7a, hsa-let-7b,and hsa-miR-486	Upregulation in ASD patients: hsa-let-7a, hsa-let-7b, and hsa-miR-486. Upregulation in maternal population: hsa-let-7a.	-	-	7
	Han	2019	China	Atrial septa of ASD patients	3	3	miR-29, miR-143/145, miR-17-92, miR-106b-25, and miR-503/424	Upregulated: miR-29 and miR-143/145. Downregulated: miR-17-92, miR-106b-25 and miR-503/424.	-	miR-29, miR-143/145 regulate focal adhesion. miR-17-92, miR-106b-25 impact TGF-beta and Bone Morphogenetic Protein signaling. MiR-503/424 influence the cell cycle and MAPK pathways. In mouse heart development, miR-29b-3p consistently upregulates, while miR-29c-3p downregulates. The miR-143/145 cluster increases, and downregulated miRNA clusters rise from E17.5 to adulthood.	6
	Jia	2022	China	Blood samples and cardiac tissue samples of familial ASD patients	5	13	miR-19 a,b	Downregulated(in vitro)	-	The c.335-1G > A mutation located at the splicing site of nkx2-5 in individuals with familial ASD has been implicated in the upregulation of PYK2 mRNA expression and phosphorylation by downregulating the expression of miR-19a/19b.	6

### 3.2. LncRNAs

LncRNAs are RNA molecules longer than 200 nucleotides lacking a protein-coding function. However, in terms of cellular physiology, lncRNAs exhibit a multifaceted impact, including the regulation of gene expression, transcription, DNA replication, and DNA repair through various mechanisms, such as signaling, decoying, scaffolding, and guiding functions [16–18]. Dysregulation of lncRNAs is linked to numerous diseases, highlighting their potential as valuable biomarkers for the timely detection of various pathological states [16]. Five lncRNAs (*STX18-AS1*, *AA709223*, *BX478947*, *HOTAIR*, and *Moshe*) are associated with ASD progression [19–22].

#### 3.2.1. *STX18-AS1*

The lncRNA, *LOC100507266*, is syntaxin 18 antisense RNA 1 (STX18-AS1). Syntaxins constitute a family of soluble N-ethylmaleimide-sensitive factor (NSF) attachment proteins primarily within the endoplasmic reticulum. Among these, syntaxin 5 and syntaxin 18 play pivotal roles in transportation between the endoplasmic reticulum and Golgi in mammals [23, 24].

The first genome-wide association study by Cordell et al. [19] on European patients with CHDs revealed an association between a region on chromosome 4p16 and the occurrence of ASD. Within this region, three specific single-nucleotide polymorphisms (SNPs) related to ASD were identified: rs6824295 (T>C), rs16835979 (A>C), and rs870142 (T>C). Among these SNPs, rs870142 demonstrated the highest level of significance. Previous studies had already linked rs870142 to the development of Wolf-Hirschhorn syndrome, a congenital disorder characterized by symptoms such as failure to thrive, intellectual disability, cleft palate, and CHDs [25]. Interestingly, these three SNPs were found to downregulate the expression of the lncRNA, *STX18-AS1*, which plays a crucial role in ASD development and resides within the 4p16 chromosome region [19]. Subsequently, Zhao et al. [26] performed a genome-wide association study on the Han Chinese population, confirming the role of these SNPs in ASD development. They also observed a high degree of linkage disequilibrium among these SNPs in their study population, with rs16835979 having the lowest p-value and remaining significant in their replication study.

Liu et al. [27] provided further evidence of the causal role of the *STX18-AS1* gene in ASD development. They elucidated several mechanisms by which this lncRNA contributed to ASD. Firstly, *STX18-AS1* has a trans-acting inhibitory effect on *NK2 Homeobox5* (*NKX2-5)* expression, a known transcription factor regulating working and conducting myocyte proliferation through the Notch pathway [27, 28]. Previous research has demonstrated the association between *NKX2-5* mutations/knockdown with ASD, atrioventricular conduction blocks, pulmonary hypertension, and hyperplastic atria, supporting its status as a downstream target of *STX18-AS1* [28]. Secondly, the study demonstrated that knocking down *STX18-AS1* resulted in a decrease in the in vitro differentiation of human embryonic stem cells into cardiomyocytes, further indicating its involvement in cell differentiation processes related to cardiac development [27].

#### 3.2.2. *AA709223 and BX478947*

Gu et al. [20] identified two lncRNAs: namely *AA709223* and *BX478947*. These two lncRNAs showed significant downregulation in the plasma of pregnant women carrying fetuses with ASD in comparison with pregnant women with normal fetuses. The findings suggest that these lncRNAs could play a crucial role in ASD development and hold potential as novel biomarkers for the prenatal diagnosis of fetal ASD.

#### 3.2.3. HOTAIR

Jiang et al. [22] found that HOX transcript antisense RNA (*HOTAIR*), located within the Homeobox C (*HOXC*) gene cluster on chromosome 12 and co-expressed with the *HOXC* gene cluster, was significantly upregulated in the plasma and cardiac tissue samples of patients with ASD compared with healthy controls. Still, their study established no correlation between the *HOTAIR* expression level and the defect size. Interestingly, among ASD cases, those with pulmonary arterial hypertension showed increased *HOTAIR* expression by comparison with those without it, although this alteration did not reach statistical significance. Jiang and colleagues also revealed that HOTAIR interacted with various proteins, including enhancer of zeste homolog1 (EZH1), EZH2, SUZ12, RE1 silencing transcription factor (REST), lysine (K)-specific demethylase 1A (KDM1A)/lysine-specific histone demethylase 1A (LSD1), and RCOR1 (CoREST). EZH2 and SUZ12 are essential enzymatic subunits of the polycomb-repressive complex 2 (PRC2), an important histone methyltransferase. This enzymatic complex is responsible for effecting methylation on lysine-27 of histone H3 (H3K27me1/2/3), which serves as an epigenetic mark to silence genes. EZH2, the integral subunit of PRC2, is the key gene-silencing regulator in embryonic stem cells and plays a vital role in developing various tissues and organs, notably the heart. It regulates the expression of cardiac transcription factors, such as *GATA4* [29, 30]. Furthermore, KDM1A (LSD1), the first known histone demethylase, was also found to interact with *HOTAIR*. This enzyme is essential to normal cardiac development and pathogenesis [31]. Overall, the study suggested that *HOTAIR* might play a significant role in the pathogenesis of ASD, possibly through its interactions with key regulatory proteins involved in cardiac development and gene expression.

#### 3.2.4. MALAT 1

Metastasis-associated lung adenocarcinoma transcript 1 (*MALAT1*) is an lncRNA consisting of over 8000 nucleotides in length. It is located on chromosome 11q13 and was first discovered in 2003 for its association with metastasis in individuals suffering from non-small cell lung cancer (32).

Subsequent research has revealed that this lncRNA also plays crucial roles in hepatic, bladder, breast, cervical, and colorectal cancers [32, 33]. In addition to its involvement in cancer, *MALAT1* regulates diverse pathophysiological processes in the cardiovascular system. Specifically, it regulates the proliferation, apoptosis, autophagy, and pyroptosis of cardiomyocytes and endothelial cells [34–37]. *MALAT1* promotes cardiomyocyte proliferation through the phosphoinositide 3-kinase/protein kinase B (PI3K/Akt) pathway [37], prevents apoptosis in endothelial cells via the Nrf2 pathway [38], and induces pyroptosis through the miR-22-NLRP3 pathway [39]. Activation of the MALAT1-miR-558-ULK1 pathway protects cardiomyocytes, contributing to cell protection and reduced apoptosis following myocardial injury [40]. Consequently, *MALAT1* is involved in the pathology of several cardiovascular diseases, including atherosclerosis, myocardial infarction, heart failure, and diabetic cardiomyopathies [36]. Recognizing the multifaceted roles of *MALAT1* in cardiovascular diseases, Li et al. [41] investigated the relationship between three SNPs (rs11227209 C>G, rs619586 A>G, and rs3200401 C>T) within *MALAT1* and CHDs. Their findings revealed that *MALAT1* rs619586 polymorphism significantly increased the risk of CHD progression, principally for the progression of ventricular septal defect, while no significant association was found in patients with ASD.

#### 3.2.5. Moshe (1010001N08ik-203)

LncRNA *1010001N08ik-203*, referred to as *Moshe* by Kim et al. [21], is one of the *GATA6* transcripts identified as an antisense transcript located upstream of *GATA6*. *Moshe* plays a crucial role in developing the primary and secondary heart fields. In comparison with established lncRNAs implicated in heart development and disease, such as *Upperhand* (NR_154048.1), *Moshe* displays elevated expression levels. To investigate this aspect, Kim and colleagues adopted two approaches. First, they found that *Moshe* bound to the promoter and enhancer regions of *NKX2-5*, resulting in increased *NKX2-5* expression. Second, knocking down *Moshe* significantly reduced *NKX2-5* expression, while *GATA6* expression remained unaffected. Notably, silencing *Moshe* was associated with increased expression of genes linked to the secondary heart field, including *Isl-1*, *Hand2*, and *Tbx2*. In contrast, there were no changes in the expression of genes associated with the primary heart field, namely *Hcn4*, *Tbx5* (T-Box transcription factor 5), and *GATA6*. Consequently, the authors suggested that *Moshe* might play a significant role in cardiac development and could have implications for ASD pathogenesis through its influence on the expression of *NKX2-5* and the regulation of genes associated with the secondary heart field.

### 3.3. miRNAs

miRNAs are short ncRNAs, consisting of around 21 to 23 nucleotides. They exert their influence by regulating post-transcriptional expression, principally through mRNA degradation and inhibiting translation. This point underscores how the disrupted expression of miRNAs can stymie the progression of different diseases, notably cardiovascular conditions [42, 43]. Several miRNAs, including hsa-miR-19a, hsa-miR-19b, hsa-miR-375, hsa-miR-29c, miR-29, miR-143/145, miR-17-92, miR-106b-25, and miR-503/424, miR-9, miR-30a, miR-196a2, miR-139-5p, hsa-let-7a, hsa-let-7b, and hsa-miR-486, have been associated with ASD development [44–48].

#### 3.3.1. Hsa-miR-19a, hsa-miR-19b, hsa-miR-375, and hsa-miR-29c

Zhu et al. [44] found that in pregnant women with an ASD fetus, three miRNAs were significantly upregulated in serum samples compared with those with a normal fetus: hsa-miR-19b, hsa-miR-375, and hsa-miR-29c.

Previous research has suggested that miR-19b-1 reduces the levels of pro-angiogenic proteins, such as fibroblast growth factor receptor 2, by inhibiting their gene expression. In addition, miR-19b-1 inhibits cell cycle progression [49]. Regarding miR-375 and miR-29c, several studies have explored their connections to the carcinogenesis process in various conditions, including colorectal carcinomas, Barrett’s esophagus, esophageal squamous cell carcinoma, and nasopharyngeal carcinoma [50–55].

Jia et al. [56] uncovered another genetic mutation, c.335-1G > A, at the splicing region of *nkx2-5* in patients with familial ASD. This splicing mutation is at the junction of *NKX2-5* intron 1 and exon 2. They also showed that the *NKX2-5* c.335-1 G > A mutation did not influence cardiomyocyte differentiation and revealed that the c.335-1 G > A mutation might lessen the expression of NKX2-5 at the protein level via *NKX2-5* nonsense-mediated mRNA degradation. Their study highlighted that this mutation, c.335-1 G > A, might upregulate the expression and phosphorylation of proline-rich tyrosine kinase 2 (PYK2) by inhibiting the expression of miR-19a/19b.

PYK2 is a key cytoskeletal protein and tyrosine kinase in the focal adhesion complex, bridging intracellular and extracellular signal transduction. It regulates cell processes like proliferation, differentiation, apoptosis, and inflammation in cardiovascular, nervous, and skeletal systems [57–59]. Activation of PYK2 induces the stress-activated protein kinase/Jun amino-terminal kinase pathway, leading to excessive cardiomyocyte apoptosis and ASD development.

#### 3.3.2. miR-139-5p

miR-139-5p, located on chromosome 11q13.4, has the potential to serve as a biomarker for various diseases. Previous studies have shown its promise as a potential cancer biomarker owing to its association with tumor proliferation, invasion, and metastasis [60]. Further, miR-139-5p could function as a diagnostic biomarker for myocardial infarction since its upregulation prompts the suppression of endothelial cell viability by inhibiting *VEGFR-1* [61].

Wang et al. [62] discovered a novel mutation, c.*1784T>C, located in the 3′UTR of the *ACTC1* gene among patients with familial isolated secundum ASD [62]. Previous studies have shown that *ACTC1* is responsible for encoding α cardiac actin, the predominant actin in the embryonic myocardium, and is a well-known gene associated with ASD development [63, 64]. They have also provided evidence that reduced expression of *ACTC1* is linked to familial and sporadic ASD through various mechanisms, including the induction of cardiomyocyte apoptosis [63, 64]. The c.*1784T>C mutation in the 3′UTR of *ACTC1* results in a new miR-139-5p target site. The binding of miR-139-5p to this newly formed response element site in the 3′UTR of *ACTC1* leads to diminished *ACTC1* expression. Consequently, this mutation in the 3′UTR of *ACTC1* may increase the risk of familial secundum ASD progression by decreasing *ACTC1* expression.

#### 3.3.3. miR-196a2

Yu et al. [45] demonstrated that in patients with sporadic ASD, the rs11614913 (T>C) SNP of miR-196a2 was associated with ASD occurrence. The homozygous CC variant of the miR-196a2 and miR-196a2 C alleles showed a negative association with ASD compared with the wild-type T allele, suggesting a protective role against ASD. Additionally, miR-196a2 rs11614913 polymorphism (T>C) can increase its expression under physiologic and pathologic conditions [45, 65]. Interestingly, this miRNA polymorphism is not associated with other CHDs, including ventricular septal defect and patent ductus arteriosus. On the other hand, previous studies have shown that the miR-196a2 rs11614913 polymorphism (T>C) is associated with an increased risk of various malignancies, such as esophageal cancer, lung cancer, and hepatocellular carcinoma [65–67]. These findings highlight the multifaceted roles of miR-196a2 and its genetic variation in different disease contexts, suggesting its potential as a significant regulatory factor in various pathophysiologic conditions.

#### 3.3.4. miR-9 and miR-30a

*TBX5* is a well-known gene crucial for developing the primary heart field, with expression observed in myocardial and pericardial tissues across all cardiac chambers in both embryonic and adult cardiac tissues. Wang et al. [46] investigated the 3′UTR region of the *TBX* gene in ASD patients. Their objective was to explore potential variations in this region that might be related to ASD through their influence on *TBX* expression. Their finding led to the discovery of a *TBX5* 3′UTR variant, rs6489956 C>T, which increased susceptibility to ASD. The CT and TT genotypes were associated with an elevated risk of ASD progression compared with the wild CC genotype. Furthermore, the T allele had a high binding affinity to two miRNAs, miR-9 and miR-30a, when compared with the C allele. As a result, this change in binding affinity reduces *TBX5* expression through both transcriptional and translational levels.

#### 3.3.5. miRNAs hsa-let-7a, miRNAs hsa-let-7b, and hsa-miR-486

The let-7 family, consisting of 12 members (let-7-a1, a2, a3, b, c, d, e, f1, f2, g, I, and miRNA-98), was one of the first mammalian miRNA families recognized as tumor suppressors. This family inhibits cell proliferation effectively by downregulating growth signaling proteins like RAS, myc family, and HMGA2. The downregulation of the hsa-let-7 family is commonly associated with the pathogenesis of numerous human cancers, making them potential early diagnostic and prognostic biomarkers for malignancies based on previous studies [68–70].

Song et al. [47] demonstrated that the role of the let-7 family extended beyond cancer, and they proposed its involvement in CHDs. In their study, 84 cardiovascular-related miRNAs were investigated in plasma samples from children with CHDs and their parents. The finding revealed that children with ASD showed a significant upregulation of hsa-let-7a, hsa-let-7b, and hsa-miR-486 compared with healthy children. Interestingly, a significant increase in the expression level of hsa-let-7a was observed only in the maternal population with ASD offspring compared to mothers with healthy offspring (gender-based comparison). All three of these miRNAs demonstrated considerable discriminatory power in distinguishing ASD patients from healthy controls, according to the receiver operating characteristic curve analysis. Notably, hsa-let-7a and hsa-let-7b exhibited the highest discriminatory power in distinguishing ASD patients from healthy controls. Moreover, in the maternal population, hsa-let-7a and hsa-let-7b displayed high accuracy in differentiating mothers with ASD children from mothers with healthy children.

#### 3.3.6. miR-29, miR-143/145, miR-17-92, miR-106b-25, and miR-503/424

Han et al. [48] observed significant downregulation of miR-29 and miR-143/145 clusters, while miR-17-92, miR-106b-25, and miR-503/424 clusters were upregulated in the atrial septum tissues of sporadic ASD patients compared with healthy controls. These miRNAs play vital roles in various signaling pathways essential for normal cardiac development and morphogenesis. For instance, miR-29 and miR-143/145 are involved in focal adhesion, miR-17-92 and miR-106b-25 are associated with the TGF-β signaling pathway, miR-17-92 is additionally related to the bone morphogenetic protein signaling pathway, and miR-503/424 clusters regulate the cell cycle and mitogen-activated protein kinase signaling pathway, influencing extracellular matrix expression. During heart development in mice, Han and colleagues examined the expression pattern of the introduced miRNAs by analyzing atrial septa from embryos at different developmental stages (E10.5, E12.5, E13.5, E14.5, E15.5, E17.5, E18.5, and E21.5). The results indicated stable upregulation of miR-29b-3p and consistent downregulation of miR-29c-3p throughout the process. Likewise, the miR-143/145 cluster showed an increasing expression trend during development, whereas the downregulated miRNA clusters exhibited a gradual increase in expression from the E17.5 stage to adulthood.

**Fig 2:**
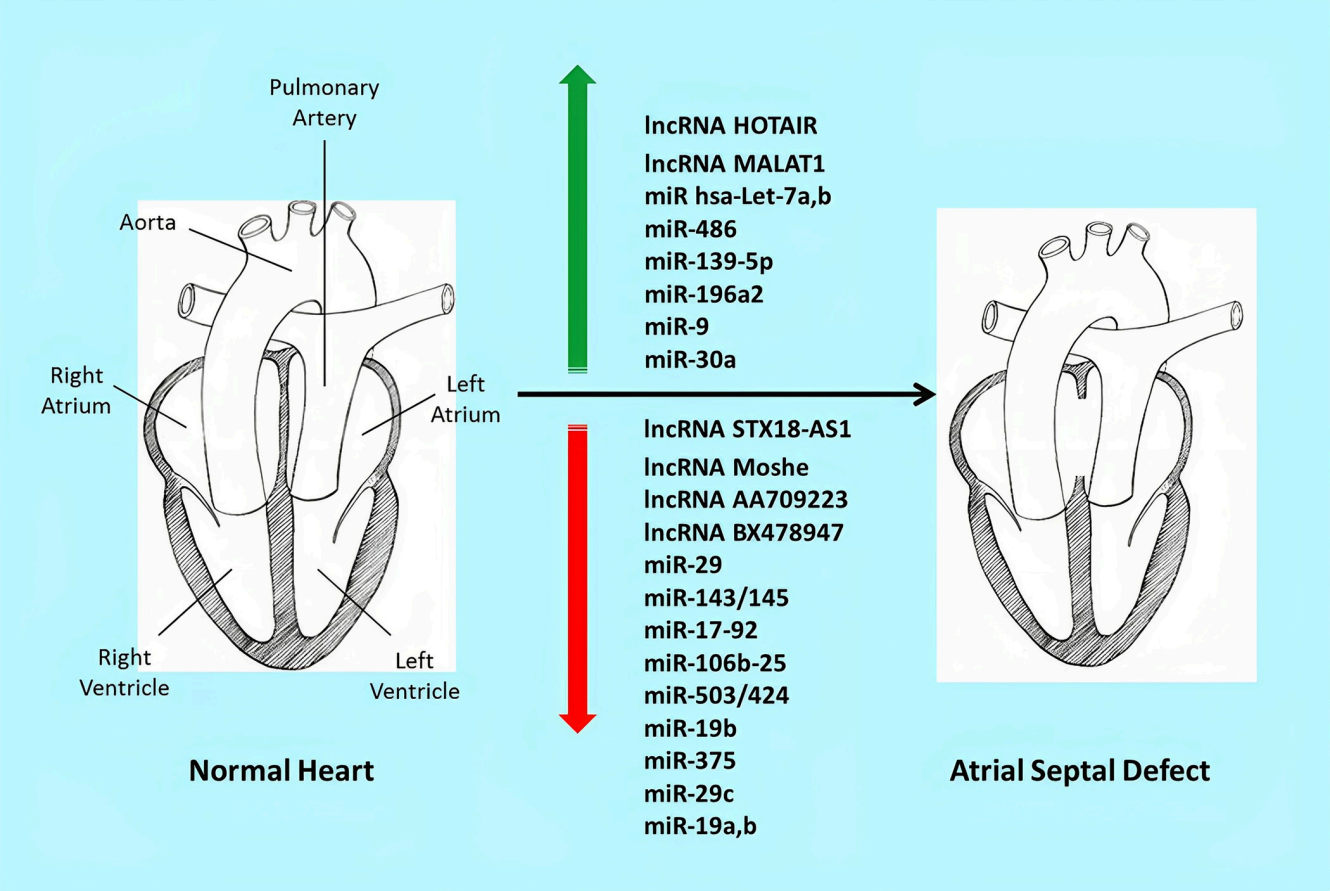
Differential expression of lncRNAs and miRNAs in ASD pathogenesis. lncRNAs: long non-coding RNAs; miRNAs: microRNAs; ASD: atrial septal defect.

Differential expressions of LncRNAs and miRNAs are shown in Fig 2.

## 4. Discussion

Since ncRNA research is a young and fast-growing field, studies on ncRNAs and CHDs are limited.

In addition, while the various roles of ncRNAs in gene expression regulation have been well established in recent years, their involvement and functionality for CHD diagnosis and prognostication remain unclear and need further investigation. This study incorporates results from thirteen peer-reviewed scientific studies. The most widely used methods for investigating ncRNAs associated with ASD in the included studies were quantitative real-time polymerase chain reaction and functional analysis using the peripheral blood samples of ASD patients.

The included studies primarily focused on miRNAs and linear lncRNAs. However, other species of ncRNAs, such as enhancer RNAs, circular RNAs, PIWI-interacting RNAs, and small interfering RNAs, have not been explored and are not discussed in this systematic review. The included studies exhibited heterogeneity in the study population, sample size, study design methods, and investigated ncRNAs. Consequently, a meta-analysis was not feasible for this study. The majority of the studies focused on known ncRNAs in ASD patients, while two studies identified novel ncRNAs. Nearly half of the included studies had a small sample size, emphasizing the need for larger samples to validate the results. A few studies confirmed their results with functional analysis, while the majority did not.

*STX18-AS1*, *AA709223*, and *BX478947* have been identified as lncRNAs whose downregulation is associated with ASD. Decreased levels of *STX18-AS1* were detected in a relatively large population of ASD patients. This downregulation interferes with normal cardiomyocyte proliferation and differentiation by affecting various pathways, including NKX2-5 [27, 28]. Downregulated *AA709223* and *BX478947* were also observed in a small population of women pregnant with ASD fetuses. Conversely, the lncRNA *HOTAIR* was upregulated in ASD patients, with a higher upregulation noted in those progressing to pulmonary arterial hypertension, suggesting a correlation with disease severity. The lncRNA *Moshe*, a regulator of mouse cardiac development and human cell differentiation into cardiomyocytes, may play a role in developing CHDs, such as ASD, by modulating the secondary heart field gene network through the regulation of NKX2-5 expression. In the Chinese population, the *MALAT1* rs619586 A>G polymorphism is associated with a minimal, non-significant increase in the risk of ASD progression, with a significant association noted in ventricular septal defect progression. This polymorphism reduces *MALAT1* expression levels.

Potential associations between *STX18-AS1*, *AA709223*, *BX478947*, and *Moshe* and other cardiovascular diseases need elucidation. In contrast, prior investigations have established a link between *HOTAIR* and *MALAT1* with coronary artery disease and heart failure. *HOTAIR* has been identified as a negative regulator of myocardial infarction in murine models, operating through the modulation of miR-519d-3p [71, 72]. Similarly, *MALAT1* has been implicated in cardiac contractility, hypertrophy, and failure, displaying correlations with miR-133 [73]. Moreover, Wang et al. [74] investigated the relationship between *MALAT1* rs619586 and the potential risks of coronary artery disease.

MiRNAs, another subclass of ncRNAs, are crucial to ASD progression. Polymorphisms in *ACTC1* and *TBX5* can change 3′UTR to target sites of hsa-miR-139-5p and has-miR-9/30a, respectively, increasing susceptibility to ASD. Additionally, the expression of hsa-let-7a, hsa-let-7b, hsa-miR-486, miR-29, miR-143/145, miR-375, and miR-29c increased in ASD patients, while miR-17-92, miR-106b-25, miR-503/424, and miR-19a expression decreased. Interestingly, some studies showed that the expression level of miR-19b could be upregulated and downregulated in ASD, potentially influenced by the type of samples. These miRNAs play their regulatory roles by affecting different pathways, such as focal adhesion, cell cycle, TGF-β, and mitogen-activated protein kinase signaling pathways.

Several of the abovementioned miRNAs have been identified as potential diagnostic and therapeutic targets in other cardiovascular diseases. For instance, miR-19a, miR-375, and miR-139-5p have diagnostic potential in acute myocardial infarction, cardiac hypertrophy, and heart failure [75–77]. MiR-375 protects against hypoxia-induced cardiac cell apoptosis through the modulation of the Nemo-like kinase pathway [76]. MiR-139-5p, implicated in myocardial infarction, inhibits endothelial cell viability by targeting VEGFR-1. Notably, miR-9 and hsa-let-7b-5p are essential to atherosclerosis and coronary artery disease development [61, 78], and miR-486 confers protection against cardiac myocardial apoptosis by targeting PTEN and FoxO1 and activating the AKT/mTOR pathway, holding promise as a therapeutic strategy for myocardial protection [79].

## 5. Strengths and limitations

This systematic review presents several salient strengths. It stands as the most comprehensive assessment of ncRNAs in ASD pathogenesis to date. Crucially, no time restrictions were imposed during the search process, which enhanced the inclusivity of relevant literature. The systematic approach to identifying studies minimizes the likelihood of overlooking pertinent research. However, certain limitations should be acknowledged. The exploration of ncRNAs in CHDs is still an emerging research field, resulting in a scarcity of information and precluding a quantitative data analysis owing to study heterogeneity and insufficient data. Moreover, the dominance of studies from China (9 out of 13) raises concerns about the generalizability of findings to other ethnicities. Additionally, the lack of in vitro and functional studies limits the depth of our knowledge in this field.

## 6. Conclusion

The existing literature features, albeit to a limited extent, studies exploring the involvement of ncRNAs in ASD, one of the most common CHDs. This study demonstrated changes in the expression of miRNAs (has-miR-19a, hsa-miR-19b, has-miR-375, hsa-miR-29c, miR-29, miR-143/145, miR-17-92, miR-106b-25, and miR-503/424, miR-9, miR-30a, miR-196a2, miR-139-5p, hsa-let-7a, hsa-let-7b, and hsa-miR-486) and lncRNAs (*STX18-AS1*, *HOTAIR*, *AA709223*, *BX478947*, and *Moshe*) related to ASD development. Investigating ncRNA roles in ASD may offer the potential to advance ncRNA-based therapeutic strategies and discover valuable biomarkers.

## Supporting information

S1 Table Detailed search queries. (DOCX)

S2 Table Quality assessment of the included studies. (DOCX)

S1 File PRISMA checklist. (DOCX)

## Abbreviations

• ASD: Atrial septal defect
• CHD: Congenital heart disease
•ACTC1: Cardiac α-actin 1 gene
• EZH: Enhancer of zeste homolog
• HOTAIR: HOX transcript antisense RNA
• KDM1A/LSD1: Lysine (K)-specific demethylase 1A/Lysine-specific histone demethylase 1A
• LncRNA: Long non-coding RNA
• MALAT1: Metastasis-associated lung adenocarcinoma transcript 1
• MiRNA: Micro RNA
• NcRNA: Non-coding RNA
• PI3K/Akt: Phosphoinositide 3-kinase/Protein kinase B
• PRC2: Polycomb-repressive complex 2
• REST: RE1 Silencing Transcription Factor
• SNP: Single-nucleotide polymorphism
• TBX5: T-Box Transcription Factor 5
• UTR: Untranslated region
• VEGFR-1: Vascular endothelial growth factor receptor-1
• PYK2: Proline-rich tyrosine kinase 2
