# Supplementary material for: Exploring the role of non-coding RNAs in atrial septal defect pathogenesis: A systematic review
Source: PLoS One. 2024 Aug 22;19(8):e0306576. doi: 10.1371/journal.pone.0306576 (PMC11340980; doi:10.1371/journal.pone.0306576)
Supplement: S1 Table — (DOCX) [file pone.0306576.s001.docx]

Supplementary Table 1. Search strategies in Pubmed, Embase, Scopus, and Web of science.

| Pubmed | ("Heart Septal Defects, Atrial"[Mesh] OR "Heart Defects, Congenital"[Mesh] OR [Defects, Congenital Heart] OR [Atrial Septal Defects] OR [Atrial Septal Defect] OR [Septal Defect, Atrial] OR [Persistent Ostium Primum] OR [Ostium Primum, Persistent] OR [Primum, Persistent Ostium] OR [Atrial Septal Defect Ostium Primum] OR [Ostium Primum Atrial Septal Defect] OR [Ostium Secundum Atrial Septal Defect] OR [Defect, Atrial Septal] OR [Defect, Congenital Heart] OR [Abnormality, Heart] OR [Heart Abnormality] OR [Congenital Heart Defect] OR [Heart, Malformation Of] OR [Malformation Of Heart] OR [Malformation Of Hearts] OR [Defects, Congenital Heart] OR [Heart Abnormalities] OR [Heart Defect, Congenital] OR [Congenital Heart Disease] OR [Congenital Heart Diseases] OR [Disease, Congenital Heart] OR [Heart Disease, Congenital] OR [Congenital Heart Defects) AND ("RNA, Circular"[Mesh] OR [circRNAs] OR [circRNAs] OR [Circular RNA] OR [Circular Intronic RNA] OR [ciRNA] OR [RNA, Circular Intronic] OR [RNAs, Circular] OR [circRNA] OR [circRNAS] OR [RNA, Closed Circular] OR "RNA, Untranslated"[Mesh] OR [Untranslated RNA] OR [npcRNA] OR [RNA, Nontranslated] OR [RNA, Non-Peptide-Coding] OR [Non-Peptide-Coding RNA] OR [RNA, Non-Protein-Coding] OR [Non-Protein-Coding RNA] OR [RNA, Non Protein Coding] OR [RNA, Non-coding] OR [Non-coding RNA] OR [RNA, Non-Coding] OR [Non-Coding RNA] OR [RNA, Non Coding] OR "MicroRNAs"[Mesh] OR [MicroRNA] OR [miRNAs] OR [miRNA] OR [siRNA OR [si-RNA OR [PiRNA] OR [pi-RNA]) |
| --- | --- |
| Embase | ('heart atrium septum defect'/exp OR 'congenital heart disease'/exp OR 'heart defects' OR 'atrial septal defects' OR 'atrial septal defect' OR 'septal defect, atrial' OR 'persistent ostium primum' OR 'ostium primum, persistent' OR 'primum, persistent ostium' OR 'atrial septal defect ostium primum' OR 'ostium primum atrial septal defect' OR 'ostium secundum atrial septal defect' OR 'heart abnormality' OR 'congenital heart defect' OR 'heart, malformation of' OR 'malformation of heart' OR 'malformation of hearts' OR 'heart abnormalities' OR 'congenital heart disease' OR 'congenital heart diseases' OR 'congenital heart defects') AND ('microrna'/exp OR 'circular ribonucleic acid'/exp OR 'small untranslated rna'/exp OR 'long untranslated rna'/exp OR 'circular ribonucleic acid' OR 'circular rna' OR 'circular intronic rna' OR 'cirna' OR 'rna, circular intronic' OR 'rnas, circular' OR 'circrna' OR 'circrnas' OR 'rna, closed circular' OR 'untranslated rna'/exp OR 'untranslated rna' OR 'npcrna' OR 'non-peptide-coding rna' OR 'non-protein-coding rna' OR 'rna, non-coding' OR 'non-coding rna' OR 'rna, non coding' OR 'microrna' OR 'mirnas' OR 'mirna' OR 'snrna*' OR 'short-interfering-ribonucleic-acid' OR 'sirna' OR 'si-rna' OR 'pirna' OR 'pi-rna') |
| Scopus | (ALL("Heart Septal Defect*") OR ALL("Congenital Heart Defect*") OR ALL("Atrial Septal Defect*") OR ALL("Atrial Septal Defect*") OR ALL("Septal Defect*") OR ALL("Persistent Ostium Primum") OR ALL("Persistent Ostium") OR ALL("Ostium Primum") OR ALL("Ostium Secundum ") OR ALL("Heart Abnormality*") OR ALL("Heart Malformation") OR ALL("Malformation Of Heart") OR ALL("Malformation Of Hearts") OR ALL("Heart Abnormalities") OR ALL("Heart Defect, Congenital")) AND (ALL("Circular RNA*") OR ALL("circRNA*") OR ALL("Circular Intronic RNA") OR ALL("ciRNA") OR ALL("RNA Circular Intronic") OR ALL("RNAs Circular") OR ALL("circRNAS") OR ALL("RNA Closed Circular") OR ALL("RNA Untranslated") OR ALL("Untranslated RNA*") OR ALL("npcRNA") OR ALL("RNA Nontranslated") OR ALL("RNA Non-Peptide-Coding") OR ALL("Non-Peptide-Coding RNA") OR ALL("Non-Protein-Coding RNA*") OR ALL("RNA Non Protein Coding") OR ALL("RNA Non-coding") OR ALL("Non-coding RNA*") OR ALL("RNA Non-Coding") OR ALL("Non-Coding RNA") OR ALL("MicroRNAs") OR ALL("MicroRNA") OR ALL("miRNAs") OR ALL("miRNA") OR ("lncRNA*") OR ("long non coding RNA*") OR ("long untranslated RNA*") OR ("siRNA"( OR ")si-RNA" (OR )"PiRNA"( OR) "pi-RNA")) |
| Web of Science | TS=("Heart Septal Defects? " or "Heart Defect? " or "Congenital Heart Defect?” or “Atrial Septal Defects? " or "Atrial Septal Defect? " or "Septal Defect? " or "Persistent Ostium Primum? " or "Ostium Primum ?" or "Ostium Secundum ? " or "Heart Abnormality? " or "Heart Malformation Of? " or "Malformation Of Heart?" or "Malformation Of Hearts? " or "Heart Abnormalities? " or "Heart Defect Congenital? " or "Congenital Heart Disease? " or "Congenital Heart Diseases?" or "Heart Disease Congenital?" or "Congenital Heart Defects") AND TS=("RNA, Circular? " or "circRNAs? " or "Circular RNA? " or "Circular Intronic RNA? " or "ciRNA? " or “RNA Circular Intronic?" or "circRNA? " or "circRNAS? " or "RNA Closed Circular?" or "RNA Untranslated?" or "Untranslated RNA?" or "npcRNA?" or "RNA Nontranslated? " or " Non-Peptide-Coding RNAs?" or "Non-Peptide-Coding RNA?" or "Non-Protein-Coding RNA? " or "RNA Non Protein Coding? " or "RNA Non-coding? " or "Non-coding RNA? " or "Non-Coding RNAs? " or "RNA Non Coding? " or "MicroRNAs? " or "MicroRNA? " or "miRNAs?" or "miRNA?"or "siRNA?" or "si-RNA?" or "PiRNA?" or "pi-RNA?" or "lncRNA?" or "lncRNAs?" or "long non coding RNA?" or "long-non-coding RNA?") |
