# Supplementary material for: Exploring the role of non-coding RNAs in atrial septal defect pathogenesis: A systematic review
Source: PLoS One. 2024 Aug 22;19(8):e0306576. doi: 10.1371/journal.pone.0306576 (PMC11340980; doi:10.1371/journal.pone.0306576)
Supplement: S2 Table — (DOCX) [file pone.0306576.s002.docx]

**Supplemental Table 1. Quality assessment of the included studies N/A: Not applicable.**

| Study | Selection |  |  |  | Comparability | Exposure |  |  | Scores |
| --- | --- | --- | --- | --- | --- | --- | --- | --- | --- |
|  | Adequate definition of cases | Representative-ness of the cases | Selection of  controls | Definition of  controls | Control for important factor | Ascertainment of exposure | Same method  to ascertain for cases and controls | Non-Response  rate | |
| Cordell, 2013 | * | * | * | - | * | - | * | * | 6 |
| Gu, 2016 | * | * | * | * | * | * | * | * | 8 |
| Jiang, 2018 | * | * | * | * | * | * | * | * | 8 |
| Li, 2018 | * | * | * | * | * | * | * | * | 8 |
| Kim, 2021 | - | - | - | - | - | - | - | - | N/A |
| Liu, 2022 | - | - | - | - | - | - | - | - | N/A |
| Zhu, 2013 | * | * | * | * | * | * | * | * | 8 |
| Wang, 2016 | * | - | - | * | * | - | * | - | 4 |
| Yu, 2016 | * | * | * | * | * | * | * | * | 8 |
| Wang, 2017 | * | * | * | * | * | * | * | * | 8 |
| Song, 2018 | * | * | * | * | * | * | * | * | 8 |
| Han, 2019 | * | - | * | * | * | * | * | * | 7 |
| Jia, 2022 | * | - | - | * | * | * | * | * | 6 |
